# *cis*-Dichloridobis[tris­(4-chloro­phen­yl)phosphane-κ*P*]platinum(II) acetonitrile monosolvate

**DOI:** 10.1107/S1600536812037166

**Published:** 2012-08-31

**Authors:** Reinout Meijboom, Leo Kirsten, Thapelo Mbhele

**Affiliations:** a Research Centre for Synthesis and Catalysis, Department of Chemistry, University of Johannesburg, PO Box 524 Auckland Park, Johannesburg, 2006, South Africa

## Abstract

The title compound, [PtCl_2_(C_18_H_12_Cl_3_P)_2_]·C_2_H_3_N, packs as monomeric units with a square-planar geometry around the Pt^II^ atom. The two tris­(4-chloro­phen­yl)phosphane ligands are coordinated in a *cis* orientation, with P—Pt—P and Cl—Pt—Cl angles of 99.36 (2) and 88.02 (2)°, respectively. In the crystal, C—H⋯N inter­actions are observed between the phenyl rings and the acetonitrile solvent mol­ecules.

## Related literature

For a review on related compounds see: Spessard & Miessler (1996 ▶). For related structures, see: Davis & Meijboom (2011 ▶); Ogutu & Meijboom (2011 ▶).
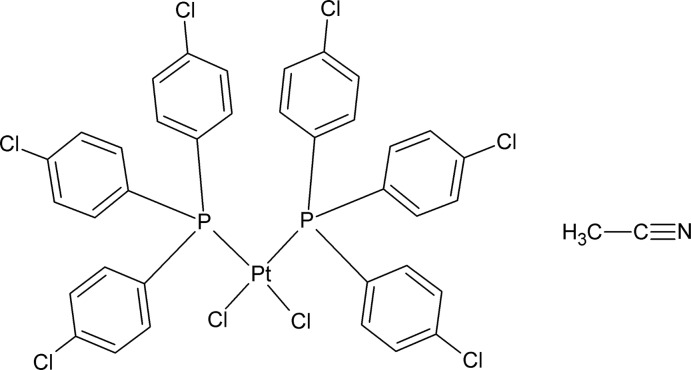


## Experimental

### 

#### Crystal data

[PtCl_2_(C_18_H_12_Cl_3_P)_2_]·C_2_H_3_N
*M* *_r_* = 1038.24
Monoclinic, 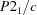

*a* = 13.3604 (16) Å
*b* = 14.4950 (16) Å
*c* = 23.007 (3) Å
β = 120.694 (2)°
*V* = 3831.3 (8) Å^3^
*Z* = 4
Mo *K*α radiation
μ = 4.34 mm^−1^
*T* = 100 K
0.45 × 0.12 × 0.08 mm

#### Data collection

Bruker X8 APEXII 4K KappaCCD diffractometer
Absorption correction: multi-scan (*SADABS*; Bruker, 2007 ▶) *T*_min_ = 0.563, *T*_max_ = 0.746
61516 measured reflections
9570 independent reflections
8248 reflections with *I* > 2σ(*I*)
*R*_int_ = 0.044

#### Refinement

*R*[*F* ^2^ > 2σ(*F* ^2^)] = 0.023
*wR*(*F* ^2^) = 0.052
*S* = 1.03
9570 reflections
452 parameters
H-atom parameters constrained
Δρ_max_ = 1.05 e Å^−3^
Δρ_min_ = −0.82 e Å^−3^

### 

Data collection: *APEX2* (Bruker, 2007 ▶); cell refinement: *SAINT-Plus* (Bruker, 2007 ▶); data reduction: *SAINT-Plus* and *XPREP* (Bruker, 2007 ▶); program(s) used to solve structure: *SHELXS97* (Sheldrick, 2008 ▶); program(s) used to refine structure: *SHELXL97* (Sheldrick, 2008 ▶); molecular graphics: *DIAMOND* (Brandenburg & Putz, 2005 ▶); software used to prepare material for publication: *WinGX* (Farrugia, 1999 ▶).

## Supplementary Material

Crystal structure: contains datablock(s) I, global. DOI: 10.1107/S1600536812037166/zq2178sup1.cif

Structure factors: contains datablock(s) I. DOI: 10.1107/S1600536812037166/zq2178Isup2.hkl

Additional supplementary materials:  crystallographic information; 3D view; checkCIF report

## Figures and Tables

**Table 1:** Selected bond lengths (Å)

Pt1—P1	2.2502 (7)
Pt1—P2	2.2525 (7)
Pt1—Cl2	2.3342 (7)
Pt1—Cl1	2.3454 (7)

**Table 2:** Hydrogen-bond geometry (Å, °)

*D*—H⋯*A*	*D*—H	H⋯*A*	*D*⋯*A*	*D*—H⋯*A*
C15—H15⋯N1^i^	0.93	2.61	3.437 (6)	148
C22—H22⋯N1	0.93	2.59	3.445 (4)	153
C56—H56⋯N1	0.93	2.68	3.523 (4)	151

Symmetry code: (i) 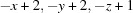.

**Fig. 1.**
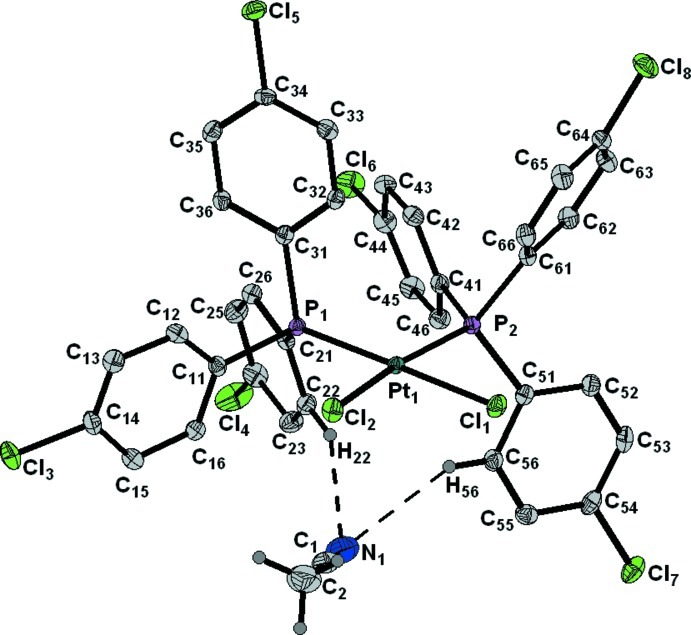
Representation of the title compound, showing the numbering scheme and displacement ellipsoids (50% probability). For the carbon rings, the first digit refers to ring number, second digit to atom in the ring. Hydrogen atoms omitted for clarity (except when involved in intermolecular interactions).

## supplementary crystallographic information

### Comment

Transition metal complexes containing phosphane, arsine and stibine ligands are widely being investigated in various fields of organometallic chemistry (Spessard & Miessler, 1996). As part of a systematic investigation involving complexes with the general formula *trans/cis*-[*MX*_2_(*L*)_2_] (*M* = Pt, Pd or Rh; *X* = halogen, Me, Ph; *L* = Group 15 donor ligand), crystals of the title compound were obtained.

[PtCl_2_(*L*)_2_] (*L* = tertiary phosphane, arsine or stibine) complexes can conveniently be prepared by the substitution of 1,5-cyclooctadiene (COD) from [PtCl_2_(COD)]. The title compound, *cis*-[PtCl_2_(C_18_H_12_Cl_3_P)_2_] crystallizes in the monoclinic spacegroup *P*2_1_/c, with the Pt atom on a general position. The *cis* coordination of the two phosphane ligands results in a distorted square-planar geometry around the Pt atom. This distortion is exemplified by the P1–Pt1–P2 bond angle of 99.36 (2) ° and the Cl1–Pt1–Cl2 bond angle of 88.02 (2) °. The Pt—P bond lengths are 2.2502 (7) and 2.2525 (7) Å, and the Pt—Cl bond lengths are 2.3342 (7) and 2.3454 (7) Å, respectively. The title compound crystallized as a solvated complex with one acetonitrile moiety per molecule.

The title compound compares well with other closely related Pt(II) complexes from the literature containing two chloro and two tertiary phosphane ligands in a *cis* geometry (Davis & Meijboom, 2011; Ogutu & Meijboom, 2011). The Pt–Cl and Pt–P bond lengths compare well with the typical values for complexes of this kind.

In the crystal structure, intermolecular C—H···N interactions are observed between phenyl rings and the acetonitrile solvent molecules.

### Experimental

Tris(4-chlorophenyl)phosphane (0.1235 g, 0.34 mmol) was dissolved in ethanol (25 cm^3^). Pt(COD)Cl_2_ (0.05 g, 0.17 mmol) was added to the solution and the mixture was allowed to reflux for 24 h. The solvent was evaporated and a white solid was obtained. Colourless crystals were obtained by recrystallization from acetonitrile, crystals suitable for a single-crystal X-ray diffraction study.

### Refinement

All H positions were calculated after each cycle of refinement using a riding model, with C—H = 0.93 Å and *U*_iso_(H) = 1.2*U*_eq_(C) for aromatic H atoms, and with C—H = 0.96 Å and *U*_iso_(H) = 1.5*U*_eq_(C) for methyl H atoms.

### Crystal data

**Table:**

[PtCl_2_(C_18_H_12_Cl_3_P)_2_]·C_2_H_3_N	*F*(000) = 2024
*M**_r_* = 1038.24	*D*_x_ = 1.800 Mg m^−^^3^
Monoclinic, *P*2_1_/*c*	Mo *K*α radiation, λ = 0.71073 Å
Hall symbol: -P 2ybc	Cell parameters from 9894 reflections
*a* = 13.3604 (16) Å	θ = 2.5–28.3°
*b* = 14.4950 (16) Å	µ = 4.34 mm^−^^1^
*c* = 23.007 (3) Å	*T* = 100 K
β = 120.694 (2)°	Plate, colourless
*V* = 3831.3 (8) Å^3^	0.45 × 0.12 × 0.08 mm
*Z* = 4	

### Data collection

**Table:**

Bruker X8 APEXII 4K KappaCCD diffractometer	9570 independent reflections
Radiation source: fine-focus sealed tube	8248 reflections with *I* > 2σ(*I*)
Graphite monochromator	*R*_int_ = 0.044
φ and ω scans	θ_max_ = 28.4°, θ_min_ = 1.7°
Absorption correction: multi-scan (*SADABS*; Bruker, 2007)	*h* = −17→17
*T*_min_ = 0.563, *T*_max_ = 0.746	*k* = −19→13
61516 measured reflections	*l* = −30→30

### Refinement

**Table:**

Refinement on *F*^2^	Primary atom site location: structure-invariant direct methods
Least-squares matrix: full	Secondary atom site location: difference Fourier map
*R*[*F*^2^ > 2σ(*F*^2^)] = 0.023	Hydrogen site location: inferred from neighbouring sites
*wR*(*F*^2^) = 0.052	H-atom parameters constrained
*S* = 1.03	*w* = 1/[σ^2^(*F*_o_^2^) + (0.0147*P*)^2^ + 7.3*P*] where *P* = (*F*_o_^2^ + 2*F*_c_^2^)/3
9570 reflections	(Δ/σ)_max_ = 0.003
452 parameters	Δρ_max_ = 1.05 e Å^−^^3^
0 restraints	Δρ_min_ = −0.82 e Å^−^^3^

### Special details

**Table:**

Experimental. The intensity data was collected on a Bruker X8 Apex II 4 K Kappa CCD diffractometer using an exposure time of 20 s/frame. A collection frame width of 0.5 ° covering up to θ = 28.4° resulted in 99% completeness accomplished.
Geometry. All s.u.'s (except the s.u. in the dihedral angle between two l.s. planes) are estimated using the full covariance matrix. The cell s.u.'s are taken into account individually in the estimation of s.u.'s in distances, angles and torsion angles; correlations between s.u.'s in cell parameters are only used when they are defined by crystal symmetry. An approximate (isotropic) treatment of cell s.u.'s is used for estimating s.u.'s involving l.s. planes.
Refinement. Refinement of *F*^2^ against ALL reflections. The weighted *R*-factor *wR* and goodness of fit *S* are based on *F*^2^, conventional *R*-factors *R* are based on *F*, with *F* set to zero for negative *F*^2^. The threshold expression of *F*^2^ > 2σ(*F*^2^) is used only for calculating *R*-factors(gt) *etc*. and is not relevant to the choice of reflections for refinement. *R*-factors based on *F*^2^ are statistically about twice as large as those based on *F*, and *R*- factors based on ALL data will be even larger.

### Fractional atomic coordinates and isotropic or equivalent isotropic displacement parameters (Å^2^)

**Table:**

	*x*	*y*	*z*	*U*_iso_*/*U*_eq_	
Pt1	0.585232 (9)	0.825075 (7)	0.384838 (5)	0.01073 (3)	
P1	0.67506 (6)	0.82097 (5)	0.49834 (3)	0.01152 (12)	
P2	0.59721 (6)	0.67465 (5)	0.36454 (3)	0.01171 (12)	
Cl1	0.48595 (6)	0.83719 (5)	0.26666 (3)	0.01794 (13)	
Cl2	0.55243 (6)	0.98251 (5)	0.38877 (3)	0.01816 (13)	
Cl3	0.86117 (7)	1.19197 (5)	0.66505 (4)	0.02474 (15)	
Cl4	1.17326 (7)	0.63784 (7)	0.66162 (4)	0.0355 (2)	
Cl5	0.32156 (6)	0.68604 (5)	0.58652 (3)	0.02180 (15)	
Cl6	0.89015 (7)	0.41498 (6)	0.61659 (4)	0.02883 (17)	
Cl7	0.89339 (6)	0.61938 (6)	0.22381 (3)	0.02344 (15)	
Cl8	0.12024 (7)	0.45956 (6)	0.24527 (4)	0.02725 (16)	
C11	0.7229 (2)	0.93258 (19)	0.54104 (12)	0.0137 (5)	
C12	0.6572 (2)	0.97964 (19)	0.56300 (13)	0.0159 (5)	
H12	0.5848	0.9568	0.5525	0.019*	
C13	0.6988 (3)	1.0603 (2)	0.60045 (13)	0.0179 (6)	
H13	0.6558	1.0907	0.6161	0.021*	
C14	0.8044 (3)	1.09449 (19)	0.61390 (13)	0.0181 (6)	
C15	0.8684 (3)	1.0529 (2)	0.58937 (14)	0.0192 (6)	
H15	0.9375	1.0791	0.5968	0.023*	
C16	0.8277 (2)	0.9711 (2)	0.55335 (13)	0.0169 (5)	
H16	0.8707	0.9417	0.5373	0.020*	
C21	0.8113 (2)	0.75688 (19)	0.54211 (13)	0.0138 (5)	
C22	0.8897 (2)	0.76648 (19)	0.51909 (13)	0.0164 (5)	
H22	0.8670	0.7981	0.4791	0.020*	
C23	1.0005 (3)	0.7295 (2)	0.55510 (14)	0.0198 (6)	
H23	1.0521	0.7361	0.5396	0.024*	
C24	1.0335 (3)	0.6825 (2)	0.61467 (14)	0.0208 (6)	
C25	0.9574 (3)	0.6697 (2)	0.63771 (14)	0.0198 (6)	
H25	0.9803	0.6367	0.6773	0.024*	
C26	0.8463 (2)	0.70648 (19)	0.60112 (13)	0.0160 (5)	
H26	0.7943	0.6974	0.6161	0.019*	
C31	0.5777 (2)	0.77817 (18)	0.52576 (13)	0.0133 (5)	
C32	0.4669 (2)	0.74861 (19)	0.47758 (13)	0.0147 (5)	
H32	0.4456	0.7478	0.4323	0.018*	
C33	0.3873 (2)	0.72032 (19)	0.49570 (13)	0.0159 (5)	
H33	0.3133	0.7006	0.4631	0.019*	
C34	0.4203 (2)	0.72202 (19)	0.56347 (13)	0.0152 (5)	
C35	0.5291 (3)	0.7526 (2)	0.61265 (13)	0.0171 (6)	
H35	0.5494	0.7541	0.6578	0.020*	
C36	0.6073 (2)	0.78100 (19)	0.59372 (13)	0.0151 (5)	
H36	0.6805	0.8022	0.6264	0.018*	
C41	0.6804 (2)	0.59576 (18)	0.43507 (13)	0.0138 (5)	
C42	0.6337 (2)	0.56608 (19)	0.47427 (13)	0.0156 (5)	
H42	0.5589	0.5841	0.4625	0.019*	
C43	0.6973 (3)	0.5104 (2)	0.53008 (14)	0.0186 (6)	
H43	0.6665	0.4919	0.5565	0.022*	
C44	0.8074 (3)	0.4829 (2)	0.54597 (13)	0.0187 (6)	
C45	0.8551 (3)	0.5093 (2)	0.50748 (14)	0.0193 (6)	
H45	0.9291	0.4894	0.5188	0.023*	
C46	0.7910 (2)	0.5658 (2)	0.45178 (13)	0.0169 (5)	
H46	0.8220	0.5837	0.4254	0.020*	
C51	0.6685 (2)	0.66152 (18)	0.31555 (12)	0.0134 (5)	
C52	0.6501 (3)	0.58608 (19)	0.27372 (13)	0.0167 (5)	
H52	0.5916	0.5439	0.2650	0.020*	
C53	0.7187 (3)	0.5735 (2)	0.24493 (13)	0.0191 (6)	
H53	0.7063	0.5234	0.2168	0.023*	
C54	0.8056 (2)	0.6365 (2)	0.25859 (13)	0.0178 (6)	
C55	0.8237 (2)	0.7135 (2)	0.29826 (13)	0.0174 (6)	
H55	0.8812	0.7561	0.3060	0.021*	
C56	0.7539 (3)	0.7259 (2)	0.32641 (13)	0.0173 (6)	
H56	0.7643	0.7777	0.3528	0.021*	
C61	0.4568 (2)	0.61567 (19)	0.32269 (13)	0.0143 (5)	
C62	0.4541 (3)	0.5194 (2)	0.31750 (13)	0.0173 (5)	
H62	0.5228	0.4871	0.3314	0.021*	
C63	0.3497 (3)	0.4716 (2)	0.29174 (14)	0.0191 (6)	
H63	0.3477	0.4078	0.2870	0.023*	
C64	0.2487 (2)	0.5204 (2)	0.27325 (14)	0.0185 (6)	
C65	0.2489 (3)	0.6155 (2)	0.27701 (14)	0.0198 (6)	
H65	0.1799	0.6473	0.2635	0.024*	
C66	0.3528 (2)	0.66336 (19)	0.30109 (13)	0.0162 (5)	
H66	0.3532	0.7275	0.3028	0.019*	
N1	0.8740 (3)	0.9362 (2)	0.40990 (16)	0.0389 (7)	
C1	0.8277 (3)	1.0050 (3)	0.39790 (17)	0.0321 (8)	
C2	0.7684 (4)	1.0938 (3)	0.3826 (2)	0.0440 (10)	
H2A	0.6899	1.0868	0.3457	0.066*	
H2B	0.8087	1.1373	0.3703	0.066*	
H2C	0.7675	1.1159	0.4217	0.066*	

### Atomic displacement parameters (Å^2^)

**Table:**

	*U*^11^	*U*^22^	*U*^33^	*U*^12^	*U*^13^	*U*^23^
Pt1	0.01178 (5)	0.01036 (5)	0.00961 (4)	0.00060 (4)	0.00513 (4)	0.00080 (4)
P1	0.0109 (3)	0.0134 (3)	0.0096 (3)	−0.0004 (3)	0.0047 (2)	−0.0002 (2)
P2	0.0129 (3)	0.0107 (3)	0.0107 (3)	0.0003 (3)	0.0054 (3)	0.0004 (2)
Cl1	0.0215 (3)	0.0192 (3)	0.0107 (3)	0.0019 (3)	0.0065 (3)	0.0031 (2)
Cl2	0.0228 (3)	0.0122 (3)	0.0180 (3)	0.0039 (3)	0.0094 (3)	0.0012 (2)
Cl3	0.0278 (4)	0.0162 (4)	0.0257 (3)	−0.0043 (3)	0.0104 (3)	−0.0080 (3)
Cl4	0.0181 (4)	0.0547 (6)	0.0332 (4)	0.0156 (4)	0.0128 (3)	0.0206 (4)
Cl5	0.0199 (3)	0.0288 (4)	0.0209 (3)	−0.0030 (3)	0.0135 (3)	0.0013 (3)
Cl6	0.0252 (4)	0.0309 (4)	0.0222 (3)	0.0049 (3)	0.0062 (3)	0.0138 (3)
Cl7	0.0183 (3)	0.0373 (4)	0.0163 (3)	0.0059 (3)	0.0100 (3)	0.0005 (3)
Cl8	0.0200 (4)	0.0290 (4)	0.0355 (4)	−0.0098 (3)	0.0162 (3)	−0.0115 (3)
C11	0.0142 (13)	0.0126 (13)	0.0113 (11)	−0.0004 (10)	0.0044 (10)	0.0003 (9)
C12	0.0138 (13)	0.0143 (13)	0.0173 (12)	0.0002 (11)	0.0062 (11)	0.0019 (10)
C13	0.0206 (15)	0.0149 (14)	0.0176 (13)	0.0030 (11)	0.0093 (12)	0.0013 (10)
C14	0.0212 (15)	0.0136 (14)	0.0132 (12)	−0.0020 (11)	0.0044 (11)	−0.0013 (10)
C15	0.0173 (14)	0.0198 (15)	0.0195 (13)	−0.0045 (12)	0.0088 (12)	−0.0025 (11)
C16	0.0185 (14)	0.0180 (14)	0.0169 (12)	−0.0017 (12)	0.0109 (11)	−0.0024 (10)
C21	0.0129 (13)	0.0137 (13)	0.0132 (12)	−0.0012 (10)	0.0054 (10)	−0.0018 (10)
C22	0.0191 (14)	0.0164 (14)	0.0142 (12)	0.0004 (11)	0.0088 (11)	0.0002 (10)
C23	0.0165 (14)	0.0244 (16)	0.0199 (13)	0.0004 (12)	0.0104 (12)	0.0014 (11)
C24	0.0143 (14)	0.0225 (16)	0.0203 (13)	0.0025 (12)	0.0049 (11)	0.0029 (12)
C25	0.0196 (14)	0.0219 (15)	0.0154 (12)	0.0029 (12)	0.0071 (11)	0.0039 (11)
C26	0.0163 (14)	0.0167 (13)	0.0149 (12)	−0.0009 (11)	0.0080 (11)	−0.0004 (10)
C31	0.0153 (13)	0.0119 (13)	0.0123 (11)	0.0015 (11)	0.0069 (10)	0.0010 (9)
C32	0.0165 (14)	0.0157 (14)	0.0104 (11)	0.0011 (11)	0.0059 (11)	0.0006 (10)
C33	0.0132 (13)	0.0155 (14)	0.0154 (12)	−0.0003 (11)	0.0047 (11)	0.0016 (10)
C34	0.0174 (14)	0.0113 (13)	0.0201 (13)	−0.0005 (11)	0.0120 (11)	0.0017 (10)
C35	0.0211 (15)	0.0175 (14)	0.0133 (12)	0.0014 (12)	0.0092 (11)	0.0008 (10)
C36	0.0146 (13)	0.0163 (14)	0.0122 (12)	−0.0012 (11)	0.0052 (10)	−0.0014 (10)
C41	0.0169 (14)	0.0100 (12)	0.0139 (12)	−0.0003 (10)	0.0074 (11)	−0.0003 (9)
C42	0.0156 (14)	0.0121 (13)	0.0186 (13)	0.0007 (11)	0.0084 (11)	0.0000 (10)
C43	0.0229 (15)	0.0169 (14)	0.0176 (13)	−0.0033 (12)	0.0115 (12)	0.0011 (11)
C44	0.0196 (14)	0.0150 (14)	0.0154 (12)	0.0020 (12)	0.0045 (11)	0.0054 (10)
C45	0.0147 (14)	0.0195 (15)	0.0196 (13)	0.0023 (12)	0.0056 (11)	0.0041 (11)
C46	0.0166 (14)	0.0170 (14)	0.0165 (12)	−0.0001 (11)	0.0080 (11)	0.0009 (10)
C51	0.0146 (13)	0.0130 (13)	0.0116 (11)	0.0027 (10)	0.0061 (10)	0.0012 (9)
C52	0.0190 (14)	0.0160 (14)	0.0139 (12)	−0.0002 (11)	0.0075 (11)	−0.0008 (10)
C53	0.0238 (15)	0.0180 (14)	0.0132 (12)	0.0026 (12)	0.0078 (12)	−0.0037 (10)
C54	0.0176 (14)	0.0236 (15)	0.0110 (12)	0.0069 (12)	0.0065 (11)	0.0035 (10)
C55	0.0148 (14)	0.0186 (14)	0.0176 (13)	−0.0005 (11)	0.0073 (11)	0.0018 (11)
C56	0.0222 (15)	0.0142 (14)	0.0162 (12)	−0.0013 (12)	0.0104 (12)	−0.0021 (10)
C61	0.0138 (13)	0.0154 (13)	0.0119 (11)	−0.0016 (11)	0.0054 (10)	−0.0019 (10)
C62	0.0175 (14)	0.0158 (14)	0.0179 (12)	0.0012 (11)	0.0086 (11)	−0.0007 (11)
C63	0.0209 (15)	0.0136 (14)	0.0212 (13)	−0.0026 (12)	0.0097 (12)	−0.0031 (11)
C64	0.0170 (14)	0.0222 (15)	0.0178 (13)	−0.0070 (12)	0.0100 (11)	−0.0059 (11)
C65	0.0148 (14)	0.0218 (15)	0.0215 (14)	0.0024 (12)	0.0083 (12)	−0.0038 (11)
C66	0.0160 (13)	0.0146 (14)	0.0159 (12)	0.0016 (11)	0.0065 (11)	−0.0011 (10)
N1	0.048 (2)	0.0372 (19)	0.0442 (18)	−0.0025 (16)	0.0331 (17)	0.0010 (14)
C1	0.043 (2)	0.031 (2)	0.0359 (18)	−0.0123 (17)	0.0299 (17)	−0.0069 (15)
C2	0.060 (3)	0.031 (2)	0.056 (2)	−0.0096 (19)	0.040 (2)	−0.0040 (17)

### Geometric parameters (Å, º)

**Table:**

Pt1—P1	2.2502 (7)	C33—H33	0.9300
Pt1—P2	2.2525 (7)	C34—C35	1.382 (4)
Pt1—Cl2	2.3342 (7)	C35—C36	1.385 (4)
Pt1—Cl1	2.3454 (7)	C35—H35	0.9300
P1—C31	1.820 (3)	C36—H36	0.9300
P1—C21	1.821 (3)	C41—C46	1.393 (4)
P1—C11	1.830 (3)	C41—C42	1.400 (4)
P2—C51	1.820 (3)	C42—C43	1.381 (4)
P2—C41	1.826 (3)	C42—H42	0.9300
P2—C61	1.826 (3)	C43—C44	1.381 (4)
Cl3—C14	1.745 (3)	C43—H43	0.9300
Cl4—C24	1.736 (3)	C44—C45	1.384 (4)
Cl5—C34	1.734 (3)	C45—C46	1.388 (4)
Cl6—C44	1.732 (3)	C45—H45	0.9300
Cl7—C54	1.742 (3)	C46—H46	0.9300
Cl8—C64	1.735 (3)	C51—C52	1.393 (4)
C11—C12	1.394 (4)	C51—C56	1.394 (4)
C11—C16	1.396 (4)	C52—C53	1.390 (4)
C12—C13	1.390 (4)	C52—H52	0.9300
C12—H12	0.9300	C53—C54	1.381 (4)
C13—C14	1.374 (4)	C53—H53	0.9300
C13—H13	0.9300	C54—C55	1.383 (4)
C14—C15	1.381 (4)	C55—C56	1.392 (4)
C15—C16	1.388 (4)	C55—H55	0.9300
C15—H15	0.9300	C56—H56	0.9300
C16—H16	0.9300	C61—C66	1.396 (4)
C21—C26	1.395 (4)	C61—C62	1.400 (4)
C21—C22	1.402 (4)	C62—C63	1.390 (4)
C22—C23	1.383 (4)	C62—H62	0.9300
C22—H22	0.9300	C63—C64	1.384 (4)
C23—C24	1.385 (4)	C63—H63	0.9300
C23—H23	0.9300	C64—C65	1.381 (4)
C24—C25	1.379 (4)	C65—C66	1.390 (4)
C25—C26	1.386 (4)	C65—H65	0.9300
C25—H25	0.9300	C66—H66	0.9300
C26—H26	0.9300	N1—C1	1.130 (5)
C31—C32	1.388 (4)	C1—C2	1.458 (6)
C31—C36	1.404 (3)	C2—H2A	0.9600
C32—C33	1.388 (4)	C2—H2B	0.9600
C32—H32	0.9300	C2—H2C	0.9600
C33—C34	1.387 (4)		
			
P1—Pt1—P2	99.36 (2)	C34—C35—H35	120.5
P1—Pt1—Cl2	88.87 (2)	C36—C35—H35	120.5
P2—Pt1—Cl2	171.49 (2)	C35—C36—C31	120.8 (3)
P1—Pt1—Cl1	176.65 (3)	C35—C36—H36	119.6
P2—Pt1—Cl1	83.69 (2)	C31—C36—H36	119.6
Cl2—Pt1—Cl1	88.02 (2)	C46—C41—C42	119.0 (2)
C31—P1—C21	108.88 (12)	C46—C41—P2	121.3 (2)
C31—P1—C11	103.33 (12)	C42—C41—P2	119.7 (2)
C21—P1—C11	100.23 (12)	C43—C42—C41	120.9 (3)
C31—P1—Pt1	111.04 (9)	C43—C42—H42	119.5
C21—P1—Pt1	116.46 (8)	C41—C42—H42	119.5
C11—P1—Pt1	115.65 (8)	C42—C43—C44	118.7 (3)
C51—P2—C41	102.20 (12)	C42—C43—H43	120.6
C51—P2—C61	110.27 (12)	C44—C43—H43	120.6
C41—P2—C61	99.83 (13)	C43—C44—C45	121.8 (3)
C51—P2—Pt1	110.17 (9)	C43—C44—Cl6	119.8 (2)
C41—P2—Pt1	119.83 (9)	C45—C44—Cl6	118.4 (2)
C61—P2—Pt1	113.57 (9)	C44—C45—C46	119.0 (3)
C12—C11—C16	118.9 (3)	C44—C45—H45	120.5
C12—C11—P1	121.0 (2)	C46—C45—H45	120.5
C16—C11—P1	120.1 (2)	C45—C46—C41	120.5 (3)
C13—C12—C11	120.7 (3)	C45—C46—H46	119.8
C13—C12—H12	119.7	C41—C46—H46	119.8
C11—C12—H12	119.7	C52—C51—C56	119.3 (2)
C14—C13—C12	118.9 (3)	C52—C51—P2	123.1 (2)
C14—C13—H13	120.6	C56—C51—P2	117.26 (19)
C12—C13—H13	120.6	C53—C52—C51	120.3 (3)
C13—C14—C15	122.0 (3)	C53—C52—H52	119.8
C13—C14—Cl3	119.1 (2)	C51—C52—H52	119.8
C15—C14—Cl3	118.9 (2)	C54—C53—C52	119.2 (3)
C14—C15—C16	118.8 (3)	C54—C53—H53	120.4
C14—C15—H15	120.6	C52—C53—H53	120.4
C16—C15—H15	120.6	C53—C54—C55	121.8 (3)
C15—C16—C11	120.6 (3)	C53—C54—Cl7	119.1 (2)
C15—C16—H16	119.7	C55—C54—Cl7	119.1 (2)
C11—C16—H16	119.7	C54—C55—C56	118.6 (3)
C26—C21—C22	118.4 (3)	C54—C55—H55	120.7
C26—C21—P1	123.3 (2)	C56—C55—H55	120.7
C22—C21—P1	118.0 (2)	C55—C56—C51	120.7 (3)
C23—C22—C21	120.9 (2)	C55—C56—H56	119.6
C23—C22—H22	119.6	C51—C56—H56	119.6
C21—C22—H22	119.6	C66—C61—C62	119.1 (3)
C22—C23—C24	119.1 (3)	C66—C61—P2	121.6 (2)
C22—C23—H23	120.4	C62—C61—P2	119.1 (2)
C24—C23—H23	120.4	C63—C62—C61	120.6 (3)
C25—C24—C23	121.5 (3)	C63—C62—H62	119.7
C25—C24—Cl4	118.7 (2)	C61—C62—H62	119.7
C23—C24—Cl4	119.8 (2)	C64—C63—C62	119.0 (3)
C24—C25—C26	119.1 (3)	C64—C63—H63	120.5
C24—C25—H25	120.5	C62—C63—H63	120.5
C26—C25—H25	120.5	C65—C64—C63	121.4 (3)
C25—C26—C21	121.1 (3)	C65—C64—Cl8	120.0 (2)
C25—C26—H26	119.5	C63—C64—Cl8	118.6 (2)
C21—C26—H26	119.5	C64—C65—C66	119.5 (3)
C32—C31—C36	118.5 (2)	C64—C65—H65	120.3
C32—C31—P1	119.19 (19)	C66—C65—H65	120.3
C36—C31—P1	122.1 (2)	C65—C66—C61	120.3 (3)
C33—C32—C31	121.3 (2)	C65—C66—H66	119.8
C33—C32—H32	119.3	C61—C66—H66	119.8
C31—C32—H32	119.3	N1—C1—C2	179.8 (7)
C34—C33—C32	118.7 (3)	C1—C2—H2A	109.5
C34—C33—H33	120.7	C1—C2—H2B	109.5
C32—C33—H33	120.7	H2A—C2—H2B	109.5
C35—C34—C33	121.6 (2)	C1—C2—H2C	109.5
C35—C34—Cl5	119.5 (2)	H2A—C2—H2C	109.5
C33—C34—Cl5	118.9 (2)	H2B—C2—H2C	109.5
C34—C35—C36	119.0 (2)		
			
P2—Pt1—P1—C31	−78.88 (10)	C33—C34—C35—C36	−0.9 (4)
P2—Pt1—P1—C21	46.48 (10)	Cl5—C34—C35—C36	179.9 (2)
Cl2—Pt1—P1—C21	−135.67 (10)	C34—C35—C36—C31	−0.6 (4)
P2—Pt1—P1—C11	163.81 (10)	C32—C31—C36—C35	1.7 (4)
Cl2—Pt1—P1—C11	−18.35 (10)	P1—C31—C36—C35	176.4 (2)
P1—Pt1—P2—C51	−124.32 (9)	C51—P2—C41—C46	19.2 (3)
P1—Pt1—P2—C41	−6.29 (11)	C61—P2—C41—C46	132.6 (2)
Cl1—Pt1—P2—C41	175.10 (11)	Pt1—P2—C41—C46	−102.8 (2)
P1—Pt1—P2—C61	111.42 (9)	C51—P2—C41—C42	−162.1 (2)
Cl1—Pt1—P2—C61	−67.19 (9)	C61—P2—C41—C42	−48.7 (2)
C31—P1—C11—C12	−22.4 (2)	Pt1—P2—C41—C42	75.8 (2)
C21—P1—C11—C12	−134.8 (2)	C46—C41—C42—C43	2.2 (4)
Pt1—P1—C11—C12	99.2 (2)	P2—C41—C42—C43	−176.5 (2)
C31—P1—C11—C16	156.3 (2)	C41—C42—C43—C44	−1.2 (4)
C21—P1—C11—C16	43.9 (2)	C42—C43—C44—C45	−0.2 (4)
Pt1—P1—C11—C16	−82.1 (2)	C42—C43—C44—Cl6	178.8 (2)
C16—C11—C12—C13	−4.3 (4)	C43—C44—C45—C46	0.7 (4)
P1—C11—C12—C13	174.4 (2)	Cl6—C44—C45—C46	−178.3 (2)
C11—C12—C13—C14	1.8 (4)	C44—C45—C46—C41	0.3 (4)
C12—C13—C14—C15	2.3 (4)	C42—C41—C46—C45	−1.7 (4)
C12—C13—C14—Cl3	−176.3 (2)	P2—C41—C46—C45	176.9 (2)
C13—C14—C15—C16	−3.8 (4)	C41—P2—C51—C52	77.3 (2)
Cl3—C14—C15—C16	174.8 (2)	C61—P2—C51—C52	−28.2 (3)
C14—C15—C16—C11	1.2 (4)	Pt1—P2—C51—C52	−154.3 (2)
C12—C11—C16—C15	2.7 (4)	C41—P2—C51—C56	−96.1 (2)
P1—C11—C16—C15	−176.0 (2)	C61—P2—C51—C56	158.5 (2)
C31—P1—C21—C26	−18.6 (3)	Pt1—P2—C51—C56	32.3 (2)
C11—P1—C21—C26	89.4 (2)	C56—C51—C52—C53	2.1 (4)
Pt1—P1—C21—C26	−145.1 (2)	P2—C51—C52—C53	−171.1 (2)
C31—P1—C21—C22	168.0 (2)	C51—C52—C53—C54	0.3 (4)
C11—P1—C21—C22	−84.0 (2)	C52—C53—C54—C55	−2.3 (4)
Pt1—P1—C21—C22	41.6 (2)	C52—C53—C54—Cl7	178.7 (2)
C26—C21—C22—C23	−2.1 (4)	C53—C54—C55—C56	1.7 (4)
P1—C21—C22—C23	171.6 (2)	Cl7—C54—C55—C56	−179.3 (2)
C21—C22—C23—C24	0.0 (4)	C54—C55—C56—C51	0.8 (4)
C22—C23—C24—C25	1.8 (5)	C52—C51—C56—C55	−2.7 (4)
C22—C23—C24—Cl4	−178.6 (2)	P2—C51—C56—C55	170.9 (2)
C23—C24—C25—C26	−1.5 (5)	C51—P2—C61—C66	−119.2 (2)
Cl4—C24—C25—C26	178.9 (2)	C41—P2—C61—C66	133.8 (2)
C24—C25—C26—C21	−0.7 (4)	Pt1—P2—C61—C66	5.0 (2)
C22—C21—C26—C25	2.5 (4)	C51—P2—C61—C62	66.5 (2)
P1—C21—C26—C25	−170.9 (2)	C41—P2—C61—C62	−40.5 (2)
C21—P1—C31—C32	−125.8 (2)	Pt1—P2—C61—C62	−169.33 (18)
C11—P1—C31—C32	128.3 (2)	C66—C61—C62—C63	−0.6 (4)
Pt1—P1—C31—C32	3.7 (2)	P2—C61—C62—C63	173.9 (2)
C21—P1—C31—C36	59.5 (3)	C61—C62—C63—C64	−2.0 (4)
C11—P1—C31—C36	−46.4 (3)	C62—C63—C64—C65	2.9 (4)
Pt1—P1—C31—C36	−171.0 (2)	C62—C63—C64—Cl8	−176.7 (2)
C36—C31—C32—C33	−1.4 (4)	C63—C64—C65—C66	−1.3 (4)
P1—C31—C32—C33	−176.3 (2)	Cl8—C64—C65—C66	178.4 (2)
C31—C32—C33—C34	0.0 (4)	C64—C65—C66—C61	−1.4 (4)
C32—C33—C34—C35	1.2 (4)	C62—C61—C66—C65	2.3 (4)
C32—C33—C34—Cl5	−179.6 (2)	P2—C61—C66—C65	−172.0 (2)

### Hydrogen-bond geometry (Å, º)

**Table:**

*D*—H···*A*	*D*—H	H···*A*	*D*···*A*	*D*—H···*A*
C15—H15···N1^i^	0.93	2.61	3.437 (6)	148
C22—H22···N1	0.93	2.59	3.445 (4)	153
C56—H56···N1	0.93	2.68	3.523 (4)	151

Symmetry code: (i) −*x*+2, −*y*+2, −*z*+1.

## References

[bb1] Brandenburg, K. & Putz, H. (2005). *DIAMOND.* Crystal Impact GbR, Bonn, Germany.

[bb2] Bruker (2007). *APEX2*, *SAINT-Plus*, *XPREP* and *SADABS* Bruker AXS Inc., Madison, Wisconsin, USA.

[bb3] Davis, W. L. & Meijboom, R. (2011). *Acta Cryst.* E**67**, m1800. 10.1107/S1600536811049269 PMC3238707 22199584

[bb4] Farrugia, L. J. (1999). *J. Appl. Cryst.* **32**, 837–838.

[bb5] Ogutu, H. & Meijboom, R. (2011). *Acta Cryst.* E**67**, m1662. 10.1107/S1600536811043789 PMC3238596 22199487

[bb6] Sheldrick, G. M. (2008). *Acta Cryst.* A**64**, 112–122. 10.1107/S0108767307043930 18156677

[bb7] Spessard, G. O. & Miessler, G. L. (1996). *Organometallic Chemistry*, pp. 131–135. New Jersey: Prentice Hall.

